# Early goal-directed therapy: what do we do now?

**DOI:** 10.1186/s13054-014-0705-8

**Published:** 2014-12-29

**Authors:** Mitchell M Levy

**Affiliations:** Rhode Island Hospital, Brown University, 593 Eddy Street, Providence, RI 02903 USA

## Abstract

The meta-analysis of early goal-directed therapy (EGDT) by Gu and colleagues in the previous issue of *Critical Care* adds to the ongoing controversy about the value of EGDT for resuscitating patients with severe sepsis and septic shock. The results of the ProCESS (protocolized care for early septic shock) and ARISE (Australasian resuscitation in sepsis evaluation) trials failed to demonstrate any benefit of EGDT or protocolized resuscitation when compared with ‘usual care’. The questions are the following: What is ‘usual’ care? What is ‘real world’ care? Do the results of a robust and well-conducted randomized controlled trial - in which many patients may be excluded for a variety of reasons - reflect the care given to patients on a daily basis in our emergency departments and intensive care units? Of course, there are no obvious answers to these questions, and many clinicians look forward to managing these patients without protocols. For now, the data do seem to support the management of patients with septic shock without mandated central lines or protocols. Does this mean we should go back to the era of ‘do whatever you want’? No consensus exists among clinicians regarding optimal hemodynamic monitoring, and to date no method has been proven to be superior. Given the amount of fluids given prior to randomization in the ProCESS and ARISE trials, ‘usual care’ appears to now include aggressive, early fluid resuscitation with at least 20 mL/kg of crystalloid and rapid administration of appropriate antibiotics. Certainly, this reflects the impact of the original trial by Rivers and colleagues and the broad-based implementation of the Surviving Sepsis Campaign Guidelines and bundles. If this continues to define ‘usual care’, then perhaps it is no longer necessary to mandate specific protocols for resuscitation, as it appears that standard sepsis management has evolved to be consistent with published protocols.

## Introduction

The meta-analysis of early goal-directed therapy (EGDT) by Gu and colleagues [1] in the previous issue of *Critical Care* adds to the ongoing controversy about the value of EGDT for resuscitating patients with severe sepsis and septic shock. The results of the ProCESS (protocolized care for early septic shock) [2] and ARISE (Australasian resuscitation in sepsis evaluation) [3] trials have been interpreted by some to sound the death knell for EGDT and the value of protocol-driven resuscitation in these critically ill patients. Both of these trials failed to demonstrate any benefit of EGDT or protocolized resuscitation when compared with ‘usual care’. However, aspects of the two trials provoke concerns about comparing them with the original study by Rivers and colleagues [4], including population severity, administration of fluids prior to randomization, and the overall mortality rate.

## Early goal-directed therapy: interpreting the recent data

In the meta-analysis by Gu and colleagues (which included the ProCESS but not the ARISE trial), a significant survival benefit from EGDT was found in seven trials - all randomized controlled trials (RCTs) - in the subgroup of ‘early’ intervention trials (that is, within 6 hours). However, by the authors’ own admission, the meta-analysis was flawed because of a high risk of bias due to the uncertain methodology of some of the trials. Though weakened by the fact that four of the included studies were published only in Chinese-language journals, this meta-analysis and another recently published study [5] raise questions about how to generalize the results of the ProCESS and ARISE trials.

The challenge for practicing clinicians is how to understand ‘usual care’ in the settings of these large RCTs. In both the ProCESS and ARISE trials, the usual care mortality was 18%. That is a remarkably low mortality for a population with septic shock. Most clinicians would ask whether this population reflects their clinical practice, given the low acuity - Acute Physiology and Chronic Health Evaluation II scores - and short intensive care unit length of stay in these two trials, and whether these results can be generalized to a sicker population seen outside the confines of an RCT in which ‘real world’ patients are excluded.

The questions are the following: What is ‘usual’ care? What is ‘real world’ care? Do the results of a robust and well-conducted RCT - in which many patients may be excluded for a variety of reasons - reflect the care given to patients on a daily basis in our emergency departments and intensive care units? Does a mortality rate of 18% represent a realistic goal in 2014? Are clinicians and hospitals prepared to be held to that mortality rate by the public and third-party payers? In the US, mandated public reporting of sepsis is now a reality in one state [6], and preparations are under way nationally to use sepsis measures for public reporting in just a few years. Will regulatory agencies hold clinicians to this mortality rate?

Of course, there are no obvious answers to these questions, and many clinicians look forward to managing these patients without protocols. For now, the data do seem to support the management of patients with septic shock without mandated central lines or protocols. However, almost two thirds of the patients in the two large RCTs were on pressors; therefore, the large majority of patients in both the ProCESS and ARISE trials were managed with the use of central lines.

Fortunately for patients and clinicians, the opportunity to address the question of what comprises ‘usual care’ will emerge over the next several years as state and national reporting takes hold [6,7]. Metrics will be collected from large populations of patients with severe sepsis and septic shock on the various modalities of monitoring available for clinicians to choose from, including physical examination. Over time, analyses of these large databases should create clarity about the use of hemodynamic monitoring and protocolized care as ‘routine care’ in guiding clinicians to improve outcomes of patients with this common illness. Meanwhile, clinicians and hospitals should continue to dedicate time and resources to establish systems to identify these patients early and begin treatment rapidly after identification. Most still agree that when it comes to sepsis, the earlier we treat, the better.

## Conclusions

Does this mean we should go back to the era of ‘do whatever you want’? No consensus exists among clinicians regarding optimal hemodynamic monitoring, and to date no method has been proven to be superior. Given the amount of fluids given prior to randomization in the ProCESS and ARISE trials, ‘usual care’ appears to now include aggressive, early fluid resuscitation with at least 20 mL/kg of crystalloid and rapid administration of appropriate antibiotics. Certainly, this reflects the impact of the original trial by Rivers and colleagues [4] and the broad-based implementation of the Surviving Sepsis Campaign Guidelines and bundles [8]. If this continues to define ‘usual care’, then perhaps it is no longer necessary to mandate specific protocols for resuscitation, as it appears that standard sepsis management has evolved to be consistent with published protocols. That would be good news indeed for patients with severe sepsis and septic shock.

## Abbreviations

ARISE: Australasian resuscitation in sepsis evaluation
EGDT: Early goal-directed therapy
ProCESS: Protocolized care for early septic shock
RCT: Randomized controlled trial
